# Intensive care unit nurses’ perceptions of night-work-related chronic fatigue and cognitive function: a cross-sectional survey

**DOI:** 10.3389/fpubh.2026.1946371

**Published:** 2026-09-04

**Authors:** Biljana Filipović, Katarina Kovačić, Adriano Friganović, Cecilija Rotim, Danijela Kundrata, Irena Kovačević, Krešimir Rotim

**Affiliations:** 1 Department of Nursing, University of Applied Health Sciences, Zagreb, Croatia; 2 Faculty of Health Sciences, University of Rijeka, Rijeka, Croatia; 3 Intensive Care Unit, General Hospital Šibenik, Šibenik, Croatia; 4 Quality Management Unit, University Hospital Centre Zagreb, Zagreb, Croatia; 5 Rotim Polyclinic, Zagreb, Croatia; 6 Quality Management Unit, General Hospital Dr. Ivo Pedišić, Sisak, Croatia

**Keywords:** chronic fatigue, healthy work environment, intensive care unit, night shift, nurses, patient safety

## Abstract

**Introduction:**

Night work disrupts circadian physiology and sleep, with documented consequences for the health and performance of nurses. In the intensive care unit (ICU), where sustained attention, rapid responses, and precision are essential to patient safety, night-work-related cognitive impairment is of particular concern for both patient and staff safety. This study examined how ICU nurses perceive the impact of night work on their cognitive functions.

**Methods:**

A cross-sectional survey was conducted using a non-probability convenience sample of 50 nurses in the one ICU in Croatia. An questionnaire based on a Croatian adaptation of the Creighton Competency Evaluation Instrument 2.0 assessed 23 clinical-competency items and 7 statements on the perceived cognitive impact of night work. Responses were analyzed descriptively and compared across sociodemographic groups using Mann–Whitney *U* and Kruskal–Wallis tests, with Benjamini-Hochberg false discovery rate (FDR) correction applied within each demographic-variable family, in SPSS 26.0 and Python.

**Results:**

Item means ranged from 3.34 to 4.86. Respondents agreed most strongly that night work can cause chronic fatigue (*M* = 4.86, *SD* = 0.35) and reduce attention (*M* = 4.38, *SD* = 0.73), weaken short-term (*M* = 4.26, *SD* = 0.94) and working memory (*M* = 4.22, *SD* = 0.79), and impair complex tasks and decision-making. Eight nominally significant subgroup comparisons emerged at raw *p* < 0.05 (e.g., chronic fatigue by age, *H*(3) = 10.02, *p* = 0.018; attention by sex, *U* = 204.00, *p* = 0.018), but none survived FDR correction (adjusted *p* ranging from 0.31 to 0.55); a post-hoc power analysis indicated the study was adequately powered only to detect large subgroup differences (minimum detectable effect at 80% power: *d* = 1.24 for the sex comparison).

**Discussion:**

These self-reported findings suggest that ICU nurses perceive night work as a potential threat to cognitive functions relevant to safe care, particularly through fatigue-related mechanisms, and underscore the need for organizational fatigue-management strategies that protect both patient safety and staff well-being, while subgroup differences by sex, age, and length of service should be treated as hypothesis-generating rather than confirmed given the sample size and multiple-comparisons correction applied.

## 1 Introduction

Continuous, around-the-clock care is a defining feature of hospital nursing, and night and rotating shifts are unavoidable for the nurses who deliver it. Night work, however, forces activity and sleep into biological times for which human physiology is poorly suited. It disrupts the circadian system and curtails sleep, and it has been linked to a broad range of adverse outcomes for nurses, including impaired sleep quality, elevated body mass index, and reduced occupational safety (1, 2). Because the circadian system coordinates sleep–wake cycles, hormone secretion, metabolism, and alertness, its chronic misalignment contributes to metabolic, cardiovascular, oncological, and mental-health burden (3, 4).

A subgroup of shift workers develops shift work sleep disorder, characterized by insomnia and excessive sleepiness arising from circadian misalignment (5). Nurses are the largest group of health-care providers and are disproportionately exposed to night work; reviews consistently link shift and night work to poorer sleep and to cardiometabolic, immune, oncological, and psychological harm (6, 7). Night work is associated with higher rates of depression among nurses (8), and with excess cardiovascular and cancer-related morbidity and mortality in large nurse cohorts (9, 10).

Beyond long-term disease risk, night work has immediate consequences for cognition. Cognition comprises the mental processes—attention, memory, language, executive function, perception, and social cognition—that underlie perception, judgment, and decision-making (11, 12). Sleep deprivation and circadian disruption reduce activity and connectivity in the dorsolateral prefrontal cortex (DLPFC), a region central to working memory and sustained attention, which provides a specific neurobiological mechanism for the selective cognitive impairment observed after night work (13, 14); this mechanism is later demonstrated directly in ICU nurses using near-infrared spectroscopy (15). Sleep deprivation and circadian disruption degrade precisely those functions that safety-critical work depends upon: sustained attention, short-term and working memory, response inhibition, and reaction time all decline, while errors of omission and commission increase (13, 14, 16). Collectively, these acute effects are captured by the established concept of work-related fatigue: a structural occupational-health problem, driven by scheduling, workload, and insufficient recovery time, that is frequently mistaken for a personal weakness or accepted as an unavoidable part of clinical work, even though healthcare professionals are rarely trained to recognize and manage it (17). For nurses, whose work demands constant vigilance and rapid, accurate judgment, such deficits are not merely a personal-health issue but a patient-safety issue.

These concerns are amplified in the intensive care unit. ICU patients are critically ill and physiologically unstable, and even brief lapses in assessment, prioritization, or response may have serious consequences. Objective studies of ICU nurses show that night shifts are accompanied by reduced prefrontal-cortex activity and cognitive performance (15), by short and fragmented sleep between consecutive shifts (18), and by circadian-driven declines in attention and working memory that raise the probability of error (19). Large surveys further report that a majority of night-shift nurses experience physiological consequences and patient-safety concerns (20), and a recent systematic review and meta-analysis confirms that shift work adversely affects nurses’ cognitive and motor performance (21). The updated Helsinki Declaration on Patient Safety in Anaesthesiology 2.0 (2025), endorsed by the European Society of Anaesthesiology and Intensive Care and national anaesthesiology societies, explicitly names fatigue and fatigue risk management systems as priorities for both patient safety and healthcare-worker well-being, underscoring that this concern extends well beyond any single specialty (17). Understanding how ICU nurses themselves perceive these effects is important: perceptions of cognitive vulnerability are not simply a proxy for objectively measured impairment but a determinant in their own right of whether nurses seek support, use fatigue countermeasures, disclose difficulties to supervisors, and adhere to safety protocols, so self-report captures a dimension of risk that objective testing alone cannot. In Croatia specifically, a recent national cross-sectional survey of critical care nurses found that healthy-work-environment standards were rarely implemented in their units and highlighted the importance of fostering healthier work environments for nurse retention and patient outcomes (22). The ICU at General Hospital Šibenik, like most Croatian hospital units, operates on 12-h shifts organized in a day-night-off–off rotation, so night-shift nurses in this setting work approximately 7–8 night shifts per month (alongside a similar number of day shifts) and are exposed to the longer end of the shift-length range associated with elevated fatigue risk in the literature (23). This focus is directly aligned with the theme of patient and medical-staff safety and a healthy work environment, in which the cognitive fitness of the workforce is a shared determinant of safe care and staff well-being; the European Society of Anaesthesiology and Intensive Care similarly identifies healthcare-worker well-being as one of four core pillars of sustainable practice, alongside emissions, energy, and supply-chain considerations, situating our findings within this broader international sustainability framework (24). However, evidence on how ICU nurses in Croatia perceive the cognitive consequences of night work remains limited, particularly in relation to patient-safety-relevant functions such as attention, working memory, and decision-making.

The general aim of this study was to examine nurses’ perceived impact of night work on cognitive functions in the intensive care unit, and to assess whether these perceptions differ by sex, age, marital status, and length of service. Specifically, the objectives were: (a) to evaluate ICU nurses’ self-perceived clinical competency during night shifts across the eight C-CEI 2.0 domains; (b) to measure the perceived impact of night work on seven cognition-relevant domains (chronic fatigue, rest, attention, short-term and working memory, complex-task performance, and decision-making); and (c) to explore whether these perceptions vary by sex, age, marital status, and length of service.

Based on this literature, we formulated three working hypotheses: (H1) perceived chronic fatigue and cognitive-impact ratings increase with age and length of service, reflecting cumulative exposure to night work, tempered by potentially reduced circadian resilience and competing non-work caregiving demands in mid-career; (H2) self-rated clinical competency during night shifts is higher among more experienced nurses, reflecting compensatory expertise; and (H3) perceived impact does not differ meaningfully by sex or marital status, which we treat as exploratory covariates given the small and unequal sex distribution in ICU nursing rather than variables with a strong *a priori* directional hypothesis. H1–H3 were tested using item-level subgroup comparisons; because these comparisons are exploratory and numerous, they are interpreted jointly with the multiple-comparisons correction described in Statistical Analysis.

## 2 Materials and methods

### 2.1 Study design and setting

This was a cross-sectional, questionnaire-based survey conducted in the Intensive Care Unit of General Hospital Šibenik, Croatia, between 26 February and 1 April 2025.

### 2.2 Participants

Eligible participants were nurses and nursing technicians employed in the ICU who worked night shifts during the study period; staff on prolonged leave during data collection or who did not perform night shifts were not eligible. Of the 60 nurses and nursing technicians employed in the ICU, all met the eligibility criteria and were invited to participate, forming a non-probability convenience sample; 50 completed the questionnaire, giving a response rate of 83.3%. Participation was voluntary and anonymous. Because this was a single-center convenience sample, findings should be generalized to other settings with caution.

### 2.3 Instrument

Data were collected with an anonymous questionnaire based on the Creighton Competency Evaluation Instrument 2.0 (C-CEI 2.0), used with the permission of the instrument’s authors (25). The instrument was translated from English into Croatian by the research team and adapted for self-assessment in the context of night work. The translation was reviewed by all authors, who are experienced nursing researchers; they assessed face validity, clarity, cultural appropriateness, and relevance to the ICU night-work context. The questionnaire comprised three parts. The first collected sociodemographic data (sex, age, marital status, and length of service). The second was a self-assessment of clinical competency during and after night work, organized into domains covering recognition of patient signs and symptoms, analysis of signs and symptoms, priority setting, seeking solutions, taking action, evaluation of outcomes, communication, and patient safety (23 items). The third part comprised seven statements on the perceived impact of night work on cognitive functions, including chronic fatigue, rest, attention, short-term and working memory, complex-task performance, and decision-making. All competency and impact items were rated on a five-point Likert scale (1 = strongly disagree, 2 = disagree, 3 = neither agree nor disagree, 4 = mostly agree, 5 = strongly agree), giving 30 rated items in total. Internal consistency in this sample was excellent for the competency subscale (Cronbach’s *α* = 0.96, 95% CI [0.95, 0.98]; item-rest correlations 0.58–0.84) and good for the impact subscale (α = 0.84, 95% CI [0.77, 0.90]; item-rest correlations 0.31–0.82, with the lowest values for the two items showing the strongest ceiling effects); α for the full 30-item instrument was 0.92. The questionnaire was administered at the beginning of a scheduled shift, rather than immediately after a night shift, to reduce the likelihood that responses reflected acute post-shift fatigue rather than nurses’ general perception of night work. The full English translation of the questionnaire is provided.

### 2.4 Statistical analysis

Analyses were performed in IBM SPSS Statistics 26.0 and cross-checked in Python (pandas, SciPy, statsmodels, Pingouin). Categorical data are presented as absolute and relative frequencies. Item responses are summarized as means and standard deviations for the whole sample and by sociodemographic subgroup. Differences between two groups were tested with the Mann–Whitney *U* test and among more than two groups with the Kruskal–Wallis test; test statistics (*U* or *H*), degrees of freedom or *Z* where applicable, and effect sizes (rank-biserial *r* for Mann–Whitney; epsilon-squared ε^2^ for Kruskal-Wallis) are reported for all comparisons. Statistical significance was set at *p* < 0.05. Because 30 items were compared across each of four demographic variables (120 tests in total), the Benjamini-Hochberg false discovery rate (FDR) procedure was applied within each demographic-variable family (30 tests per family) to control for multiple testing; FDR correction was chosen over a single Bonferroni correction across all 120 tests because Bonferroni is markedly conservative for exploratory, correlated, item-level comparisons of this kind. We additionally attempted a multivariable ordinal logistic regression of the attention item on sex, age group, and length-of-service group to adjust the sex comparison for potential confounding; this model did not converge to a stable solution because all six male respondents rated the item at the ceiling value with zero variance (quasi-complete separation), so we report this bivariate comparison with corresponding caution rather than an unstable adjusted estimate. A post-hoc power analysis (independent-samples framework, *α* = 0.05, two-sided) indicated that the study was adequately powered only to detect large effects: for the sex comparison (*n* = 6 vs. 44), achieved power was 0.07, 0.20, and 0.44 for small (*d* = 0.20), medium (*d* = 0.50), and large (*d* = 0.80) effects respectively, and the minimum effect size detectable at 80% power was *d* = 1.24; for a best-case balanced two-group comparison (25 vs. 25), the minimum detectable effect at 80% power was *d* = 0.81.

### 2.5 Ethics statement

This study was approved by the Ethics Committee of the General Hospital of Šibenik-Knin County, Šibenik, Croatia (Class: 007–10/25–01/9; Reg. No. 2182-1-50-11-25-1; dated 25 February 2025). All participants provided written informed consent prior to completing the questionnaire. Participation was voluntary, and anonymity was guaranteed: questionnaires carried no direct or indirect identifiers linking responses to individual nurses. Completed data were stored on password-protected, access-restricted institutional devices accessible only to the research team, in accordance with the hospital’s institutional data-protection procedures and Regulation (EU) 2016/679 (GDPR); because responses were fully anonymous and contained no personal identifiers, the study fell outside the stricter GDPR provisions applicable to identifiable personal data. The study was conducted in accordance with the Declaration of Helsinki.

## 3 Results

### 3.1 Sample characteristics

The sample comprised 50 ICU nurses, of whom 88% were women and 12% men, aged between 20 and over 50 years. Most were married (68%). One participant reported length of service as free text (“37 years”) rather than selecting a category; this response was recoded into the “>20 years” category (37 > 20), so length-of-service data are complete for all 50 respondents, with 15 participants (30% of the total sample) reporting more than 20 years of service. Full sample characteristics are shown in Table 1.

**Table 1:** Sociodemographic characteristics of the participants (*n* = 50).

Variable	Category	*n*	%
Sex	Female	44	88
	Male	6	12
	Other/ Prefer not to say	0	0
Age (years)	20–30	15	30
	31–40	20	40
	41–50	9	18
	> 50	6	12
Marital status	Married	34	68
	Single	13	26
	Divorced	2	4
	Widowed	1	2
Length of service	<5 years	8	16
	6–10 years	12	24
	11–20 years	14	28
	>20 years	16	32
	Missing	0	0

*n*, number of participants; %, percentage. Percentages are calculated using the total sample (*n* = 50). One participant reported length of service as free text (“37 years”); this was recoded into the >20-years category, so no length-of-service data are missing.

### 3.2 Perceived impact of night work

Across the 30 items, mean ratings ranged from 3.34 to 4.86 out of 5 (Table 2). Agreement was strongest for the statement that night work can cause chronic fatigue (4.86 ± 0.35), followed by reduced attention at work and in private life (4.38 ± 0.73), weakened short-term memory (4.26 ± 0.94), weakened working memory (4.22 ± 0.79), and insufficient time to rest before the next shift (4.22 ± 0.91). Perceived impairment of complex-task performance (4.00 ± 1.09) and slower decision-making (3.98 ± 1.08) were also endorsed. Within the competency self-assessment, nurses rated their ability to implement evidence-based interventions (4.16), adhere to patient-safety principles (4.14), and recognize patient signs and symptoms (≈4.0–4.1) highly, while the lowest-rated item overall was demonstrating reflective practice (3.34 ± 0.98).

**Table 2:** Mean and standard deviation of responses to the 30 questionnaire items.

Statement — “During night work, the nurse is able to…”/ perceived impact statement	M	SD
Recognizing patient signs and symptoms		
Recognize relevant subjective symptoms and signs	4.10	0.91
Recognize relevant objective symptoms and signs	4.08	0.78
Assess the patient’s physical environment	4.08	0.88
Assess the social determinants of health	3.74	0.90
Recognize deviations in relevant symptoms and signs	4.04	0.78
*Analyzing signs and symptoms*		
Analyze subjective patient data, signs, and symptoms	3.84	0.89
Analyze objective patient signs and symptoms	3.90	0.76
Identify the cause(s) of the clinical problem	3.80	0.88
Setting priorities		
Identify expected outcomes	3.70	0.86
Prioritize the required interventions	4.04	0.78
Seeking solutions and taking action		
Generate solutions	3.58	0.81
Use technology in patient care	3.90	0.84
Implement evidence-based interventions	4.16	0.87
Review/reassess relevant signs	3.86	0.90
Evaluating outcomes		
Evaluate interventions against expected outcomes	3.70	0.81
Modify expected outcomes, assessments, and/or interventions	3.52	0.76
Demonstrate reflective practice	3.34	0.98
Communication and documentation		
Communicate professionally with colleagues	3.80	0.99
Communicate therapeutically with patients	3.78	1.04
Document clearly, concisely, and accurately	3.66	1.12
Integrate information technology to support decision-making	3.36	0.90
Patient safety and advocacy		
Adhere to patient safety principles	4.14	0.88
Advocate for the patient	3.66	1.00
Perceived impact of night work on cognition		
Night work can cause the onset of chronic fatigue	4.86	0.35
Insufficient time to rest before the next shift	4.22	0.91
Reduces attention at work and in private life	4.38	0.73
Can weaken short-term memory	4.26	0.94
Can weaken working memory	4.22	0.79
Can hinder performance of more complex tasks	4.00	1.09
Can contribute to slower decision-making	3.98	1.08

Values are means and standard deviations on a 1–5 Likert scale. Item wording is abbreviated; full wording is given.

### 3.3 Differences by sociodemographic characteristics

**Figure 1:**
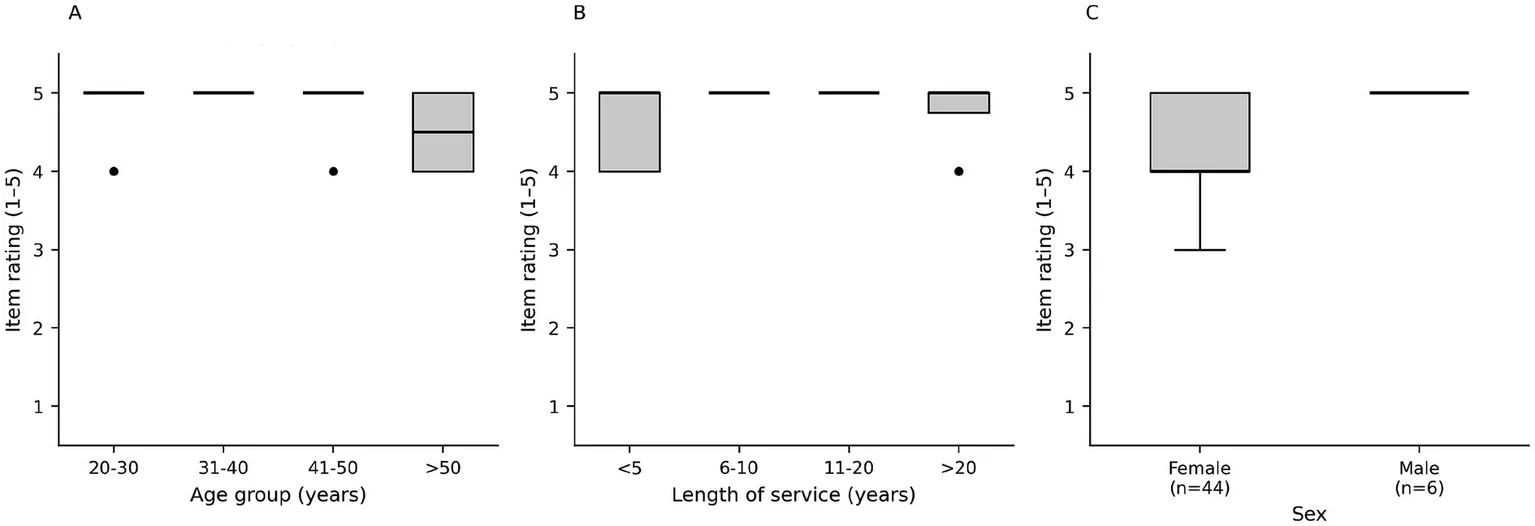
Perceived night-work impact by sociodemographic subgroup (boxplots from raw response data; *n* = 50). **(A)** Chronic fatigue onset ratings by age group. **(B)** Chronic fatigue onset ratings by length of service. **(C)** Attention-reduction ratings by sex. Boxes show the interquartile range (IQR) and median (line); several boxes are compressed to a single line because of strong ceiling effects (most ratings clustered at 4–5 out of 5). None of the three subgroup differences shown survived Benjamini-Hochberg FDR correction (Table 3).

Most comparisons across sex, age, marital status, and length of service were not statistically significant. Eight item-level comparisons reached nominal significance at raw *p* < 0.05; these are summarized with complete test statistics and effect sizes in Table 3. In exploratory subgroup analyses, the statement that night work can cause chronic fatigue was rated highest among nurses aged 31–40 years (mean 5.0; Kruskal-Wallis *H*(3) = 10.02, *p* = 0.018, ε^2^ = 0.15) and differed by length of service (*H*(3) = 9.32, *p* = 0.025, ε^2^ = 0.14). Perceived reduction in attention at work and in private life differed by sex (Mann–Whitney *U* = 204.00, *Z* = 2.36, *p* = 0.018, *r* = 0.33), with a borderline difference by age (*H*(3) = 7.85, *p* = 0.049, ε^2^ = 0.11); mean ratings were higher among men (5.0, with zero variance—all six men rated this item at the ceiling) than women (4.3), and among nurses aged 31–50 years (mean 4.6). The sex-related finding should be interpreted with particular caution: only six men participated, all rated the item identically, and an ordinal regression adjusting for age and service did not converge (quasi-complete separation), so no stable adjusted estimate could be obtained (see Statistical Analysis). For the competency items, older nurses rated themselves higher in recognizing relevant subjective symptoms and signs (*H*(3) = 8.32, *p* = 0.040, ε^2^ = 0.12), more experienced nurses rated themselves higher in identifying the cause of the clinical problem (*H*(3) = 8.28, *p* = 0.040, ε^2^ = 0.11), and both greater age (*H*(3) = 8.85, *p* = 0.031, ε^2^ = 0.13) and longer service (*H*(3) = 8.68, *p* = 0.034, ε^2^ = 0.12) were associated with higher self-ratings for evaluating interventions against expected outcomes. After applying Benjamini-Hochberg FDR correction within each demographic-variable family (30 tests per family), none of these eight comparisons remained significant (adjusted *p* ranging from 0.31 to 0.55; Table 3); we therefore report them descriptively as hypothesis-generating rather than as confirmed subgroup differences. Figure 1 illustrates the age, service, and sex patterns for the two items with the strongest raw associations (chronic fatigue and attention).

**Table 3:** Statistically significant differences in item ratings by sociodemographic variable.

Item	Variable	Test statistic	*p*	Effect size	*p* (adj.)
Chronic fatigue onset	Age	*H*(3) = 10.02	0.018^*^	ε^2^ = 0.15	0.307
Reduces attention	Sex	*U* = 204.00, *Z* = 2.36	0.018^*^	*r* = 0.33	0.547
Chronic fatigue onset	Length of service	*H*(3) = 9.32	0.025^*^	ε^2^ = 0.14	0.405
Evaluate interventions vs. expected outcomes	Age	*H*(3) = 8.85	0.031^*^	ε^2^ = 0.13	0.307
Evaluate interventions vs. expected outcomes	Length of service	*H*(3) = 8.68	0.034^*^	ε^2^ = 0.12	0.405
Recognize subjective symptoms/signs	Age	*H*(3) = 8.32	0.040^*^	ε^2^ = 0.12	0.307
Identify cause(s) of clinical problem	Length of service	*H*(3) = 8.28	0.040^*^	ε^2^ = 0.11	0.405
Reduces attention	Age	*H*(3) = 7.85	0.049^*^	ε^2^ = 0.11	0.307

H = Kruskal-Wallis test statistic (df in parentheses); U = Mann–Whitney *U* statistic; Z = standard normal deviate for the Mann–Whitney comparison; ε^2^ = epsilon-squared effect size (Kruskal-Wallis); *r* = rank-biserial effect size (Mann–Whitney). Only comparisons reaching nominal statistical significance (*p < 0.05, uncorrected) are shown; all other comparisons across sex, age, marital status, and length of service were non-significant. p (adj.) = *p*-value after Benjamini-Hochberg false discovery rate correction applied within each demographic-variable family (30 item-level tests per family). No comparison remained significant after FDR correction (all adjusted *p* > 0.05); findings should therefore be interpreted as hypothesis-generating.

## 4 Discussion

In this cross-sectional survey, ICU nurses perceived night work as a relevant potential threat to the cognitive functions that underpin safe patient care. The dominant finding was near-unanimous agreement that night work can cause chronic fatigue (4.86 ± 0.35), together with strong endorsement of reduced attention and weakened short-term and working memory. These perceptions are consistent with domains that objective studies have shown to be vulnerable to sleep loss and circadian disruption—sustained attention, working memory, and reaction time (13, 14).

The salience of chronic fatigue is consistent with the literature identifying overtime, consecutive shifts, long (predominantly 12-h) shifts, short inter-shift recovery, and high weekly hours as major predictors of fatigue in nurses (23). Framing this finding within the broader concept of work-related fatigue (17) is useful precisely because it is a structural, system-level phenomenon rather than an individual failing: the near-unanimous endorsement observed here suggests that fatigue is the default experience of night work in this unit rather than an idiosyncratic vulnerability of a few individuals.

Although the present findings are based on self-report, they are consistent with objective studies of ICU nurses showing night-shift-related changes in sleep and cognitive performance. Durán-Gómez and colleagues found that ICU nurses showed reduced dorsolateral prefrontal-cortex reactivity and poorer verbal-fluency performance after night shifts (15). Hirsch Allen and colleagues objectively measured markedly shortened sleep between consecutive night shifts in ICU nurses (18), and Moosavi and colleagues showed that circadian instability was associated with poorer attention and working memory and a higher likelihood of error during night shifts (19). At scale, Alsharari and colleagues reported that the large majority of night-shift nurses experienced physiological consequences and patient-safety concerns (20), and a recent review confirmed the consistent link between shift or night work and impaired sleep and health among medical staff (6). More broadly, the shift-work literature indicates that self-reported fatigue and cognitive-impact ratings show moderate, not perfect, agreement with objective measures such as actigraphy-derived sleep loss or psychomotor vigilance task (PVT) lapses: nurses tend to be reasonably accurate at detecting the presence of impairment but less accurate at gauging its magnitude, partly because fatigue itself can degrade metacognitive self-monitoring (21). Our results add the nurses’ own perspective to this evidence base and suggest that these perceptions are broadly consistent with, though not a precise substitute for, objectively measured effects.

A noteworthy secondary pattern was that older and more experienced nurses rated their competency—recognizing symptoms, identifying the cause of clinical problems, and evaluating interventions—more highly, even while perceiving greater fatigue, although none of these subgroup differences survived correction for multiple comparisons (see Results) and should be read as hypothesis-generating. If replicated, this pattern would be compatible with a compensatory-expertise mechanism, in which accumulated clinical experience builds automaticity, pattern recognition, and more efficient allocation of cognitive resources that partially offset the performance costs of night work on familiar, well-rehearsed tasks (26). This compensatory capacity is unlikely to be uniformly protective, however: the broader expertise literature suggests it is weakest precisely for novel, high-uncertainty, or rapidly evolving clinical situations, where fatigue-related decline is least likely to be masked by experience. Nevertheless, older healthcare professionals may also be more susceptible to the effects of fatigue because of age-related changes in sleep architecture and reduced sleep quality, which could offset any compensatory advantage of experience (26); our cross-sectional, self-report design cannot distinguish between these competing mechanisms. Self-assessed competency may also reflect greater confidence rather than objectively superior performance. Fatigue nonetheless remains a leading source of nursing error, including in medication administration (27), underscoring that experience should not be relied upon to offset inadequate rest.

These findings have practical implications for patient and staff safety. Because work-related fatigue is fundamentally a structural problem, it should be addressed primarily through structural interventions rather than through individual willpower or coping strategies. Formal Fatigue Risk Management Systems (FRMS)—data-driven, continuously monitored systems for identifying and mitigating fatigue-related risk—are already mandatory in other high-risk, safety-critical industries such as aviation, and their adaptation to healthcare has been specifically proposed as a means of protecting both clinician well-being and patient safety (28). Our findings support concrete organizational fatigue-management measures consistent with this structural approach: scheduled or strategic naps during night shifts, structured sleep-hygiene education programs, limiting consecutive shifts to a maximum of 12 h, and ensuring a minimum 11-h inter-shift rest interval, consistent with EU Working Time Directive 2003/88/EC. A recent systematic review of shift-work interventions in nurses reports meaningful quantified benefits for measures of this kind, including 20–35% reductions in fatigue scores with strategic napping and 15–40% improvements in sleep-quality scores with optimized shift planning (29). Situating cognitive fitness within a healthy work environment, and within a formal FRMS, reframes night-work fatigue from an individual burden to a shared structural safety concern that warrants system-level action.

### 4.1 Limitations

Several limitations should be considered. The study was cross-sectional and used a non-probability convenience sample from a single center (*n* = 50), which limits generalizability and precludes causal inference. Outcomes were based on self-perception rather than objective neurocognitive testing, and may be subject to recall and social-desirability bias; administering the questionnaire at shift start rather than immediately post-shift reduces but does not eliminate the possibility that responses were shaped by acute fatigue or anticipatory anxiety about the upcoming shift. The competency instrument was a Croatian adaptation of the C-CEI 2.0 that showed excellent-to-good internal consistency in this sample (*α* = 0.96 and 0.84 for the two subscales) but was not formally revalidated (e.g., confirmatory factor structure) in this setting, and some items were used as self-report rather than observed performance. A post-hoc power analysis indicated the study was adequately powered only to detect large subgroup effects (minimum detectable effect at 80% power: *d* = 1.24 for the sex comparison, *d* = 0.81 for a balanced two-group comparison), so non-significant findings should be interpreted as inconclusive rather than as evidence of no difference. The uneven distribution of some subgroups (six men, all of whom rated the attention item identically, and few widowed or divorced respondents) further limited statistical power and precluded a stable multivariable-adjusted estimate: an ordinal logistic regression adjusting the sex comparison for age and length of service did not converge because of quasi-complete separation. Eight item-level subgroup comparisons reached nominal significance (raw *p* < 0.05) but none survived Benjamini-Hochberg FDR correction applied within each demographic-variable family; these findings should therefore be interpreted as hypothesis-generating rather than confirmed. In particular, the subgroup pattern for chronic fatigue-potentially reflecting cumulative shift-work exposure alongside competing domestic and caregiving demands in mid-career - and the higher mean attention-reduction rating among men are both based on small, unadjusted subgroup comparisons and should not be read as robust age- or sex-specific effects. Future research should use larger, multicenter samples with adequate power for multivariable-adjusted subgroup analysis, and should combine self-report with objective measures of sleep and cognition, such as wrist actigraphy and the Psychomotor Vigilance Task (PVT), administered before and after night shifts.

### 5 Conclusion

ICU nurses showed near-unanimous agreement that night work causes chronic fatigue (*M* = 4.86, *SD* = 0.35 out of 5, the highest-rated item in the 30-item instrument), alongside strong endorsement of reduced attention, weakened short-term and working memory, and impaired complex-task performance and decision-making—the cognitive domains most directly relevant to safe, time-critical ICU care. Subgroup differences by age, length of service, and sex were observed at the descriptive level but did not survive correction for multiple comparisons and were constrained by limited statistical power; they should be treated as hypothesis-generating and require confirmation in larger, multicenter, adequately powered studies that pair self-report with objective measures such as actigraphy and the Psychomotor Vigilance Task. These findings nonetheless support the need for organizational fatigue-management and scheduling strategies - including capped consecutive shifts, adequate inter-shift rest, and structured napping - as part of patient-safety and healthy-work-environment initiatives, and are relevant to policy discussions around night-work provisions in Croatia’s Labour Act and the minimum daily rest requirements of EU Working Time Directive 2003/88/EC. They are consistent with, and add nurse-reported evidence to, recent international policy efforts to address night-shift-related harm, including the 2024 American Academy of Nursing consensus recommendations on night-shift nurses (30), the American Nurses Association’s 2026 position statement on nurse fatigue (31), and The Joint Commission’s Sentinel Event Alert on health-care worker fatigue and patient safety (32).

## Data Availability

The original contributions presented in the study are included in the article/supplementary material, further inquiries can be directed to the corresponding author.
